# Facial Skin Quality Improvement Using Profhilo®: A Three-Dimensional VECTRA® Image Analysis in a South American Patient

**DOI:** 10.7759/cureus.108389

**Published:** 2026-05-06

**Authors:** Felipe Gomes Dallepiane, Patricia Colett, Morgana Dalsant Trombetta, James Olding, Alessandra Kuhn Dall Magro

**Affiliations:** 1 Department of Oral and Maxillofacial Surgery, Faciallmed Center, Passo Fundo, BRA; 2 Department of Oral and Maxillofacial Surgery, Interface Aesthetics, London, GBR

**Keywords:** aesthetic procedures, facial aging, hyaluronic acid, skin rejuvenation, three-dimensional imaging

## Abstract

Facial aging is a multifactorial process that involves changes in the skin, soft tissues, musculature, and bone structure, resulting in laxity, loss of contour definition, and alterations in skin texture. This case report describes the treatment of a 57-year-old South American female patient whose primary complaint was facial aging, using the biorestructuring agent Profhilo® (IBSA Farmaceutici Italia Srl, Lodi, Italy) in combination with three-dimensional analysis via VECTRA® 3D (Canfield Scientific, Inc., Parsippany, NJ, USA). The therapeutic protocol consisted of two sessions, spaced four weeks apart, with the product applied at strategic points according to the Bio Aesthetic Points protocol, aiming to stimulate collagen production, promote deep hydration, and restore facial architecture. Pre- and post-treatment evaluation with VECTRA® 3D allowed the objective measurement of wrinkles, pores, texture, skin uniformity, and facial contours. Global improvements in firmness, hydration, texture, and contour definition were observed, demonstrating the bioremodeling and biostimulating effects of Profhilo®, with natural and progressive results. The findings from this case indicate that two sessions of Profhilo® can promote significant skin regeneration and facial rejuvenation, highlighting its applicability in postmenopausal South American populations. Moreover, this report emphasizes the utility of Profhilo® as a minimally invasive alternative for facial rejuvenation and reinforces the role of VECTRA® 3D as an objective and precise tool for the three-dimensional evaluation of aesthetic outcomes.

## Introduction

Contemporary facial aesthetics has increasingly focused on minimally invasive approaches aimed at restoring skin quality and facial harmony [1-3]. In this context, facial aging is understood as a complex three-dimensional process that involves changes across multiple anatomical layers. This phenomenon encompasses modifications in muscular, osseous, and adipose tissues, as well as cutaneous alterations such as loss of elasticity, skin thinning, and redistribution of facial fat [4-7]. These cumulative transformations significantly impact facial aesthetics and the perception of aging, motivating the development of minimally invasive techniques aimed at restoring facial harmony and youthfulness [8].

Among these approaches, hyaluronic acid (HA)-based filler injections are widely recognized as the gold standard for facial rejuvenation. Among the available products, Profhilo® (IBSA Farmaceutici Italia Srl, Lodi, Italy) stands out as an effective and safe option for restoring skin vitality and firmness, due to its high biocompatibility and the absence of chemical reagents in its formulation [9]. The product is an intradermal medical device composed of hybrid cooperative complexes of HA (HyCoCos), obtained through NAHYCO® technology (IBSA Farmaceutici Italia Srl, Lodi, Italy), an innovative thermal process that eliminates the use of any chemical reagents [10].

The hybrid complexes present in Profhilo® have demonstrated the ability to protect high-molecular-weight HA (H-HA), reducing its degradation by up to eightfold. Concurrently, low-molecular-weight HA (L-HA) is gradually released, stimulating tissue regeneration without triggering a significant inflammatory response, thereby contributing to a controlled biostimulatory effect [9]. In addition, the high concentration of HA (64 mg per 2 mL syringe) and its specific rheological properties, such as viscosity and elasticity, were specifically designed to promote remodeling and support of facial adipose tissue [11].

Therefore, the aim of the present study is to report the clinical case of a postmenopausal South American female patient who sought treatment for complaints related to facial aging, such as skin laxity, a tired appearance, and loss of contour definition following significant weight reduction. Follow-up was conducted with the aid of three-dimensional VECTRA® 3D technology (Canfield Scientific, Inc., Parsippany, NJ, USA), allowing an objective and detailed analysis of the facial changes achieved.

## Case presentation

The patient included in this case report was informed both verbally and in writing about the procedure and signed an informed consent form. The patient was a 57-year-old woman who sought treatment with the primary complaint of facial aging, characterized by skin laxity, a tired appearance, and loss of contour definition following significant weight reduction. She expressed interest in preventive strategies aimed at maintaining facial harmony and a youthful appearance.

**Figure 1:**
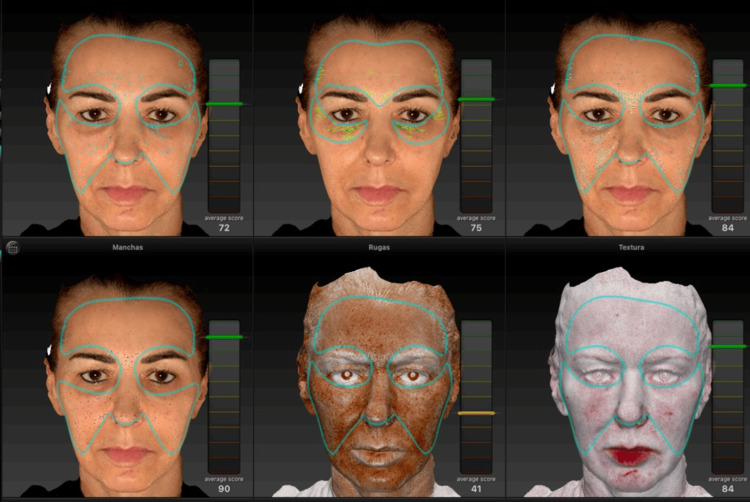
Pre-treatment three-dimensional facial analysis performed using the VECTRA® 3D system, assessing spots, wrinkles, texture, pores, brown spots, and erythematous areas through scores automatically generated by the software.

**Figure 2:**
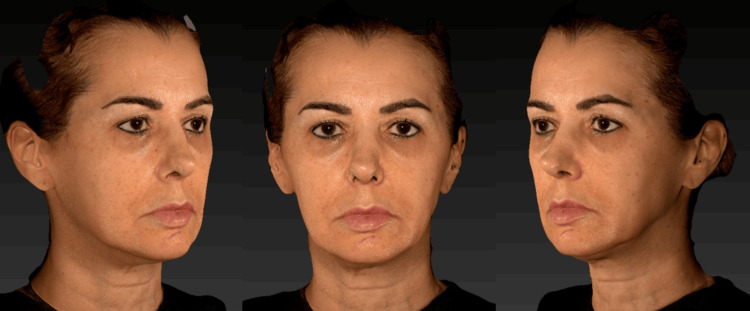
Frontal and right and left lateral images of the patient before the initiation of Profhilo® treatment.

After a detailed clinical examination and three-dimensional facial analysis (Figure 1) performed using the VECTRA® 3D system, a personalized and sequential therapeutic plan was developed, taking into account the anatomical, functional, and structural aspects of the face. The objective was to restore facial architecture and promote global facial rejuvenation (Figure 2).

Therapeutic protocol

The treatment was carried out in two sessions, with intervals planned according to the objectives of each phase and the time required for tissue regeneration. A high-fluency HA-based facial bioremodeller developed using NAHYCO® technology was used. This formulation allows wide tissue diffusion and extracellular matrix remodeling without a volumizing effect, acting naturally on the dermal structure.

First session

The initial objective was to stimulate collagen production, promote deep hydration, and improve skin texture and radiance. The injectable product (Profhilo®) was administered subcutaneously at strategic points, following the Bio Aesthetic Points (BAP) protocol, which was developed to ensure the homogeneous diffusion and uniform stimulation of collagen and elastin.

A total of 2 mL of Profhilo® was used, with 1 mL administered per side of the face. Each injection point received 0.2 mL, resulting in five injection points per hemiface. The product was applied in anatomically safe regions, including the zygomatic protuberance located 2 cm lateral to the outer canthus of the eye, the nasal base positioned 1.5 cm from the base along the midpupillary line, the pretragal region situated 1 cm below the anteroinferior margin of the tragus, the chin located 1.5 cm lateral to the midline, and the mandibular angle positioned 1 cm above the angle of the mandible.

**Figure 3:**
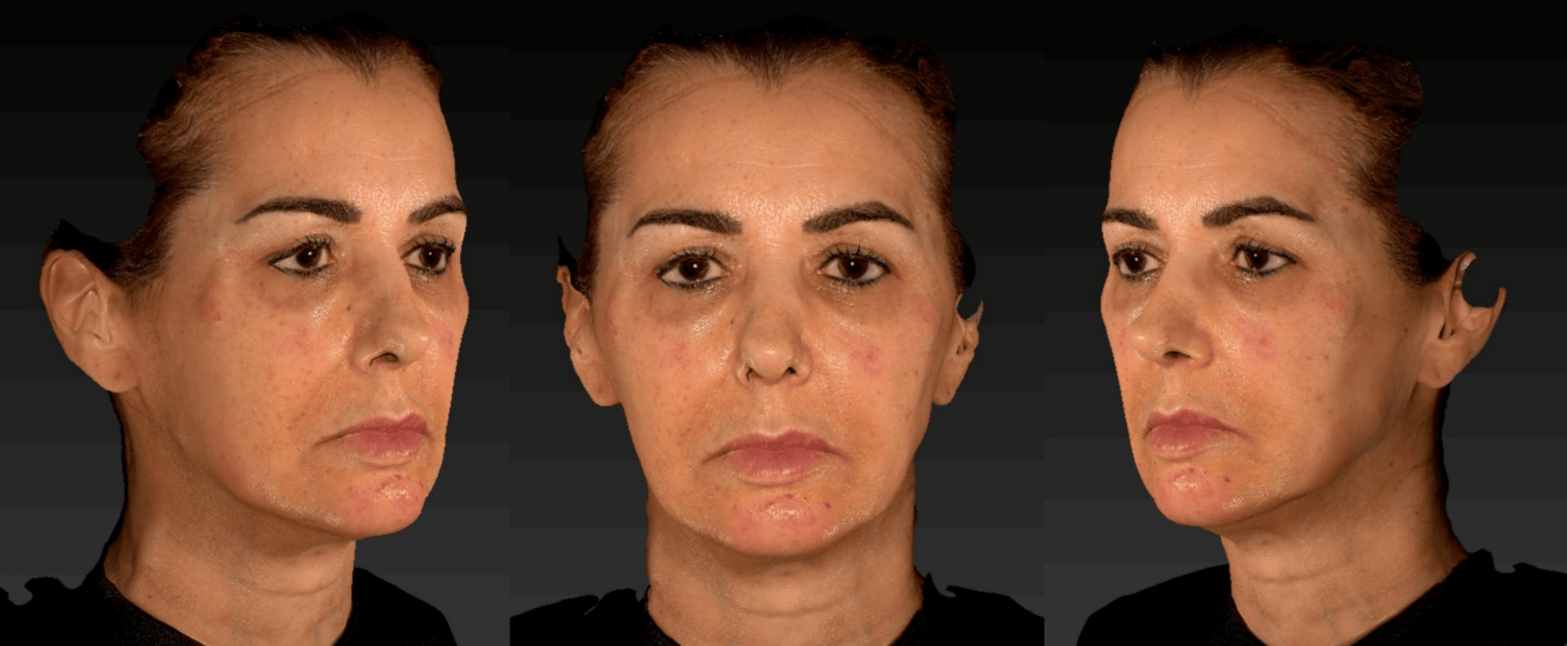
Immediate clinical appearance after the first Profhilo® application, showing mild erythema and good tissue integration, with no signs of complications.

During the application, the patient reported moderate pain (7/10) on the visual analog scale (VAS). Immediately after the procedure, the clinical appearance was documented and evaluated both visually and using the VECTRA® 3D system (Figure 3). Mild to moderate erythema and edema were observed at the injection sites, with no signs of ischemia, palpable nodules, or focal product accumulation. Small ecchymoses were identified at isolated points. Three-dimensional images confirmed homogeneous diffusion of Profhilo® within the dermal layers, with no perceptible irregularities. The patient received post-procedure instructions and was informed about the possibility of gradual resolution of erythema and edema over the subsequent weeks. No systemic adverse events were reported.

Second session and follow-up

**Figure 4:**
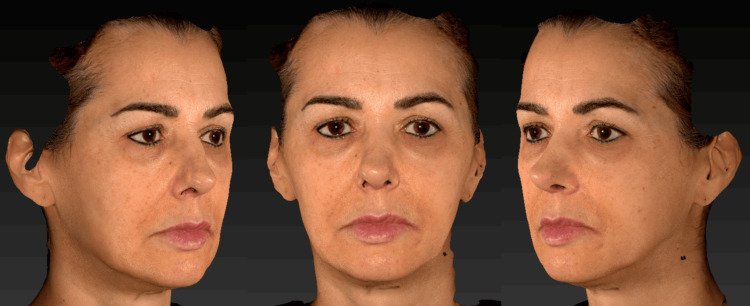
Clinical appearance 30 days after the first session, demonstrating improvement in skin texture and luminosity.

The second session was performed 30 days after the first, following the same application protocol. The patient reported mild pain and satisfactory immediate recovery, expressing satisfaction with the initial outcome (Figure 4).

**Figure 5:**
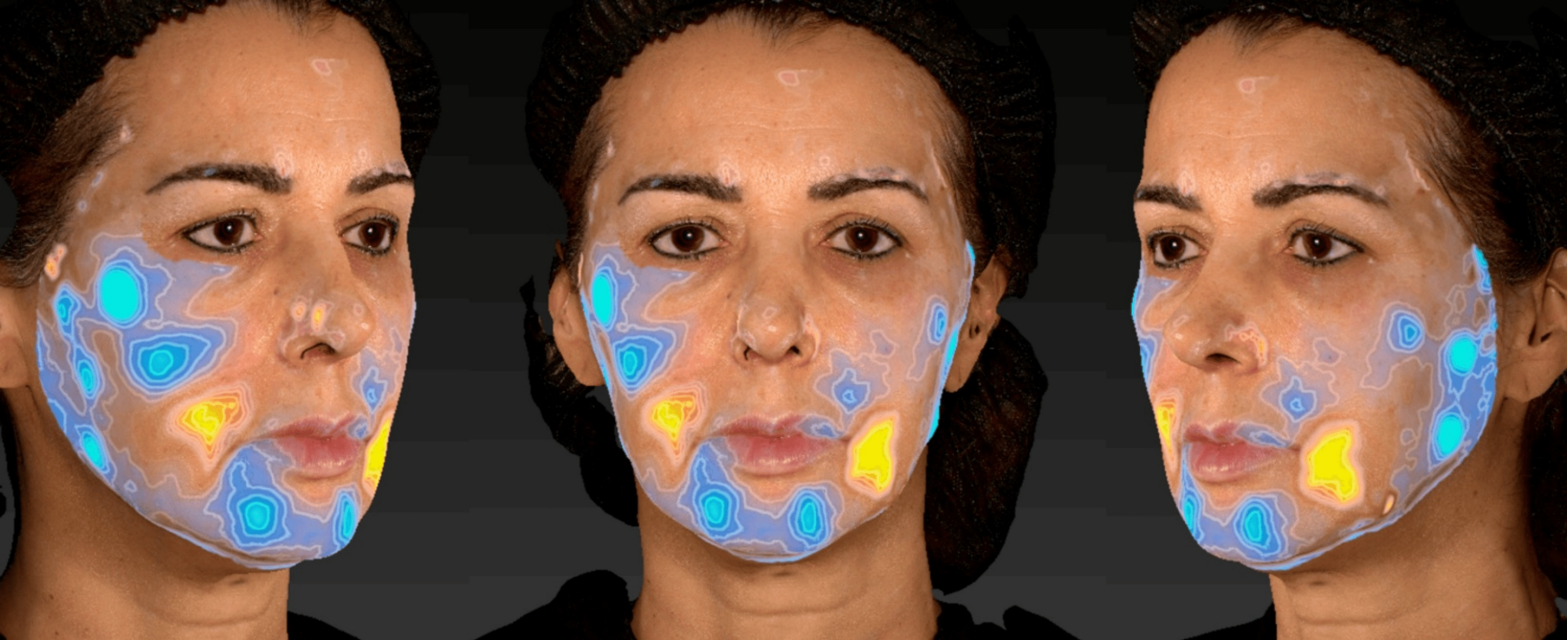
Immediate post-treatment appearance after the second application, demonstrating uniform product distribution as assessed by the VECTRA® 3D system.

The images obtained with the VECTRA® 3D system in the immediate post-treatment period after the second application (Figure 5) demonstrated the homogeneous diffusion of the product within the dermal layers, with no irregularities, confirming the high spreading capacity of Profhilo® due to NAHYCO® technology.

**Figure 6:**
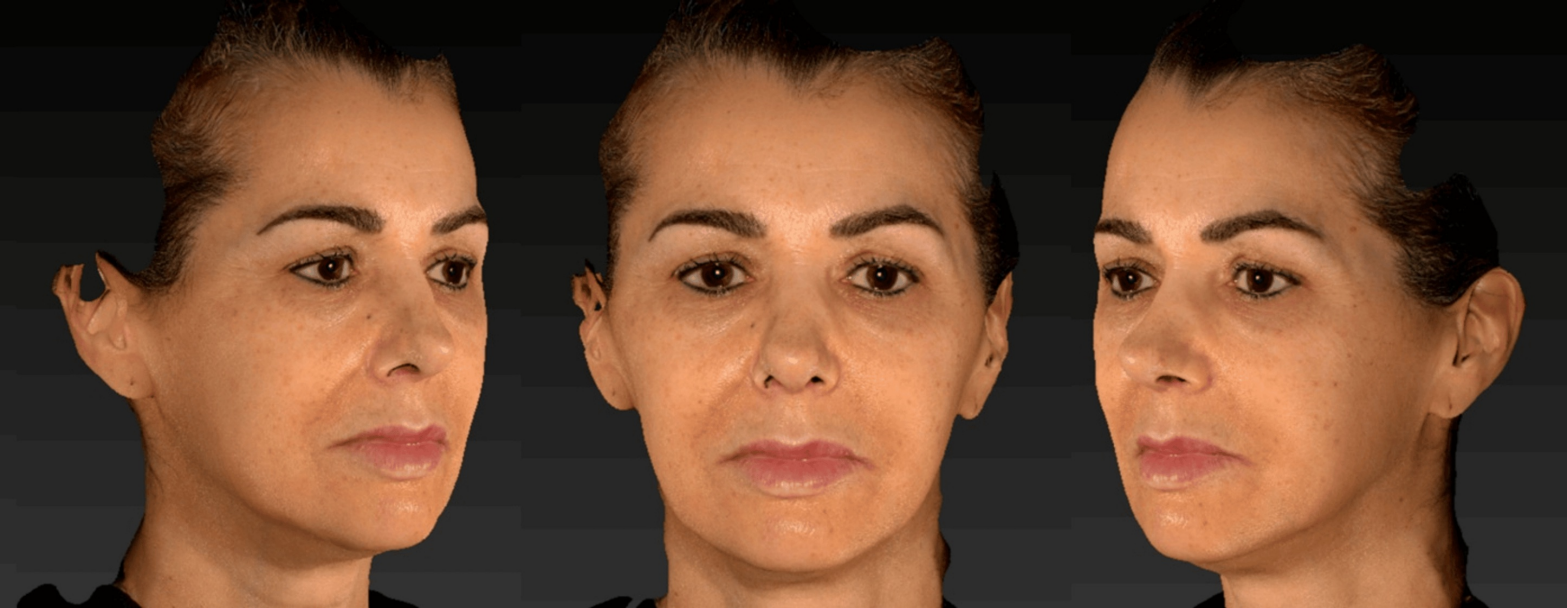
Clinical follow-up at 60 days, demonstrating improvement in skin firmness, hydration, and facial contour.

At the 60-day follow-up (Figure 6), progressive improvement in skin firmness and elasticity was observed, along with redefinition of facial contours, particularly in the malar and mandibular regions. The patient reported a high level of satisfaction with the treatment, and no adverse events were reported.

**Figure 7:**
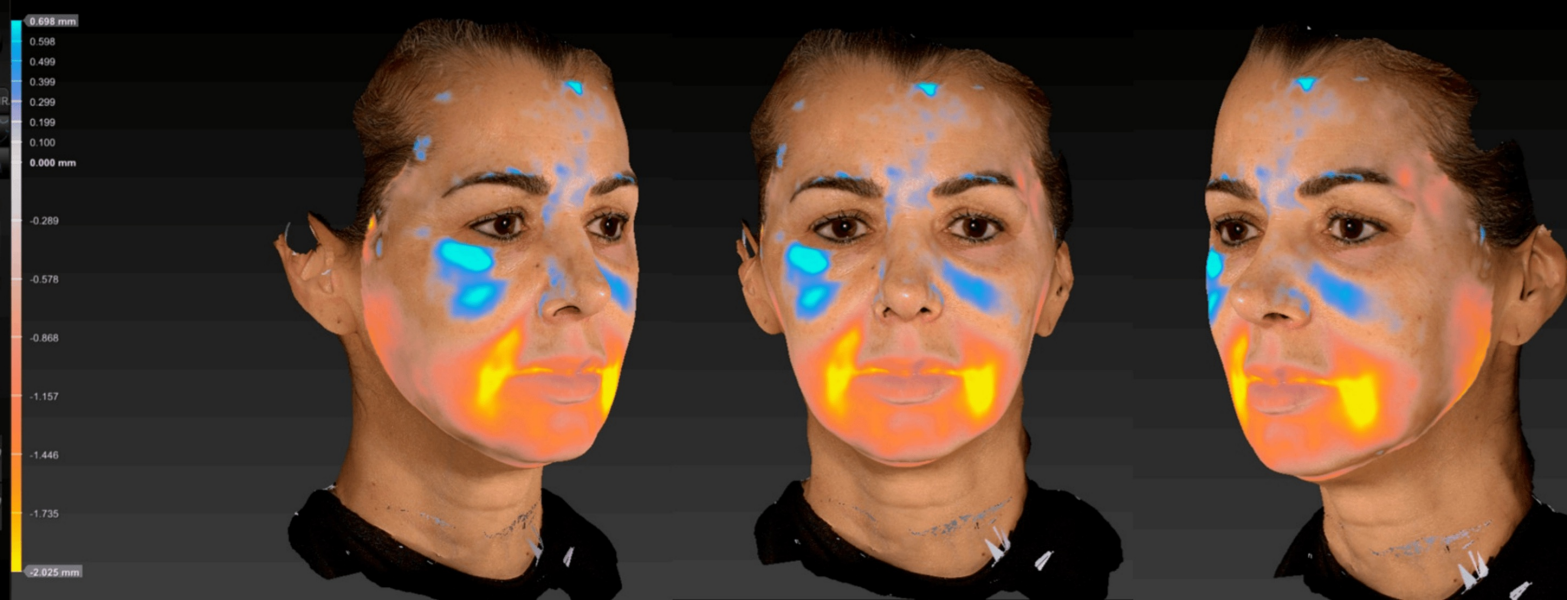
VECTRA® 3D analysis at 60 days, demonstrating an overall improvement in skin texture, color uniformity, and a reduction in wrinkles and pores.

Comparative three-dimensional analysis performed using the VECTRA® 3D system (Figure 7) demonstrated an objective reduction in wrinkles and pores, improvement in skin texture and uniformity, and a decrease in erythematous and pigmented areas. These findings reinforce the bioremodeling and biostimulatory effects of Profhilo®, demonstrating an overall improvement in skin quality and natural, progressive facial rejuvenation.

## Discussion

This study presents a case report of a South American patient who underwent treatment with Profhilo®, a HA-based bioremodeller developed to restore skin vitality and firmness, notable for its high biocompatibility and favorable safety profile [9]. Given that facial aging is a complex process involving interdependent changes across all anatomical layers, including the skin, adipose compartments, musculature, and underlying bone structure [12], understanding these transformations is essential for selecting the most appropriate therapeutic strategy. In this context, three-dimensional planning using the VECTRA® 3D system enabled a comprehensive and precise facial analysis, allowing the identification of priority treatment areas and the development of an individualized therapeutic plan.

HA is widely used worldwide to attenuate skin changes associated with aging [13]. Within this group, Profhilo® represents a significant innovation. Due to its distinctive rheological properties and high fluidity, the product demonstrates homogeneous tissue integration and provides more natural results when compared with other dermal fillers [14]. In the present study, these attributes were reflected in an overall improvement in skin firmness, hydration, and texture, highlighting the bioremodeling effect and the effectiveness of Profhilo® in restoring skin quality.

Similar results were reported by Cheng et al., who demonstrated that two sessions of Profhilo®, administered at a four-week interval, promote skin regeneration and significant improvement in facial skin quality in Asian patients [15]. These findings corroborate the results of the present clinical case, showing that the same protocol applied to a South American patient produced progressive and natural facial rejuvenation effects, thereby reinforcing the applicability and effectiveness of Profhilo® across different populations.

The use of the VECTRA® 3D system in this case report proved to be particularly useful for the objective analysis of clinical outcomes. Three-dimensional assessment allowed the measurement of morphological and texture changes, demonstrating a reduction in wrinkles and pores, improved skin uniformity, and a decrease in erythematous and pigmented areas. These quantitative findings reinforce subjective clinical observations and highlight the potential of VECTRA® 3D as a complementary tool for the scientific documentation of aesthetic outcomes [16,17]. Although few studies have systematically explored its use in the validation of aesthetic procedures, its incorporation appears promising for standardizing and improving the assessment of clinical outcomes in facial aesthetics.

Despite the positive results observed in this case, some limitations should be considered. This is an isolated case report, without a control group and with short-term follow-up, which limits the generalizability of the findings and the assessment of the durability of the observed effects. Future studies with larger sample sizes, prospective designs, longitudinal follow-up, and standardized quantitative methods are required to confirm and expand the evidence regarding the clinical performance of Profhilo® and the applicability of VECTRA® 3D as an objective analysis tool in facial aesthetics.

In addition to the limitations mentioned above, some considerations regarding the material and patient selection should be taken into account. Profhilo® demonstrates high biocompatibility and favorable diffusion; however, its non-crosslinked formulation results in temporary effects and does not provide structural support, which may limit its indication in cases requiring volumization. No significant adverse events were observed in this case; nevertheless, HA-based injectables may be associated with complications such as edema, bruising, nodules, and delayed inflammatory reactions. Furthermore, its use should be approached with caution in patients with systemic or autoimmune conditions due to the limited available evidence and the potential for unpredictable biological responses. The selection of a postmenopausal patient was based on typical intrinsic aging characteristics, which represent the main indication for this treatment. Younger patients or those with systemic or autoimmune conditions may respond differently, and current evidence in these populations remains limited.

## Conclusions

The present case demonstrates that the use of Profhilo® associated with three-dimensional facial analysis using the VECTRA® 3D system represents a reliable approach for managing skin laxity and enhancing overall facial skin quality. The application of NAHYCO® hybrid technology following a standardized injection protocol resulted in consistent, visible, and quantifiable improvements, as confirmed by objective three-dimensional assessment. This approach not only supported tissue remodeling and facial rejuvenation but also contributed to a high level of patient satisfaction, reinforcing the clinical relevance of this treatment strategy.
